# *Pleurotus sajor-caju* (Fr.) Singer β-1,3-Glucanoligosaccharide (Ps-GOS) Suppresses RANKL-Induced Osteoclast Differentiation and Function in Pre-Osteoclastic RAW 264.7 Cells by Inhibiting the RANK/NFκB/cFOS/NFATc1 Signalling Pathway

**DOI:** 10.3390/molecules29092113

**Published:** 2024-05-02

**Authors:** Purithat Rattajak, Aratee Aroonkesorn, Carl Smythe, Rapepun Wititsuwannakul, Thanawat Pitakpornpreecha

**Affiliations:** 1 Division of Health and Applied Science (Biochemistry), Faculty of Science, Prince of Songkla University, Hat-Yai, Songkhla 90110, Thailand; boomboompuri@gmail.com (P.R.); aratee.a@psu.ac.th (A.A.); 2 Center for Natural Rubber Latex Biotechnology Research and Innovation Development, Prince of Songkla University, Hat-Yai, Songkhla 90110, Thailand; rapepun.w@psu.ac.th; 3 Department of Biomedical Science, University of Sheffield, Sheffield S10 2TN, UK; c.g.w.smythe@sheffield.ac.uk

**Keywords:** glucanoligosaccharide, *Pleurotus sajor-caju*, osteoclastogenesis, osteoporosis

## Abstract

Edible grey oyster mushroom, *Pleurotus sajor-caju*, β (1,3), (1,6) glucan possesses a wide range of biological activities, including anti-inflammation, anti-microorganism and antioxidant. However, its biological activity is limited by low water solubility resulting from its high molecular weight. Our previous study demonstrated that enzymatic hydrolysis of grey oyster mushroom β-glucan using *Hevea* β-1,3-glucanase isozymes obtains a lower molecular weight and higher water solubility, *Pleurotus sajor-caju* glucanoligosaccharide (Ps-GOS). Additionally, Ps-GOS potentially reduces osteoporosis by enhancing osteoblast–bone formation, whereas its effect on osteoclast–bone resorption remains unknown. Therefore, our study investigated the modulatory activities and underlying mechanism of Ps-GOS on Receptor activator of nuclear factor kappa-Β ligand (RANKL) -induced osteoclastogenesis in pre-osteoclastic RAW 264.7 cells. Cell cytotoxicity of Ps-GOS on RAW 264.7 cells was determined by the 3-(4,5-dimethylthiazol-2-yl)-2,5-diphenyl-2H-tetrazolium bromide (MTT) assay and its effect on osteoclast differentiation was determined by tartrate-resistant acid phosphatase (TRAP) staining. Additionally, its effect on osteoclast bone-resorptive ability was detected by pit formation assay. The osteoclastogenic-related factors were assessed by quantitative reverse transcriptase polymerase chain reaction (qRT-PCR), Western blot and immunofluorescence. The results revealed that Ps-GOS was non-toxic and significantly suppressed the formation of mature osteoclast multinucleated cells and their resorption activity by reducing the number of TRAP-positive cells and pit formation areas in a dose-dependent manner. Additionally, Ps-GOS attenuated the nuclear factor kappa light chain-enhancer of activated B cells’ P65 (NFκB-P65) expression and their subsequent master osteoclast modulators, including nuclear factor of activated T cell c1 (NFATc1) and Fos proto-oncogene (cFOS) via the NF-κB pathway. Furthermore, Ps-GOS markedly inhibited RANK expression, which serves as an initial transmitter of many osteoclastogenesis-related cascades and inhibited proteolytic enzymes, including TRAP, matrix metallopeptidase 9 (MMP-9) and cathepsin K (CTK). These findings indicate that Ps-GOS could potentially be beneficial as an effective natural agent for bone metabolic disease.

## 1. Introduction

β-Glucans are polymers of D-glucose that consisted of various glycosidic linkages including β-(1,3), β-(1,3)/(1,4), β-(1,3)/(1,6) and β (1,4) bonds [1,2]. They are widely distributed in many natural sources such as bacteria, yeast, mushrooms and higher plants [3,4,5]. Fungal β (1,3), (1,6)-glucan is the major cell wall component of fungi, mushrooms and yeast, which displays several biological activities such as anti-inflammation, reduction and control of blood glucose and anti-osteoarthritis [6,7,8]. Interestingly, it also possesses biological activity against osteoporosis, which is characterized by microdamage of bone architecture resulting from an excessive osteoclast–bone resorption over osteoblast–bone formation. Currently, the attenuation of osteoclastogenesis has been considered an effective therapeutic strategy for osteoporosis.

Osteoclastogenesis is the complex process that involves differentiation and proliferation of precursor cells of myeloid origin to multinucleated mature osteoclast. This multistep process requires macrophage colony-stimulating factor (M-CSF) and RANKL. M-CSF is essential for the proliferation and survival of osteoclast precursors, whereas RANKL is required for osteoclast differentiation and function [9,10,11]. Interaction of RANKL with RANK receptor stimulates adapter protein tumour necrosis factor receptor-related factor 6 (TRAF6), which enhances many downstream signalling cascades, including the NFκB, mitogen-activated protein kinase (MAPK), c-Jun N-terminal kinase (JNKs) and calcium calmodulin pathways [12]. Among these signalling pathways, the RANKL-induced NFκB pathway serves as a pivotal mechanism for osteoclastogenesis. NF-κB1/2 double-deleted (dKO) mice display extensive osteopetrosis due to unsuccessful osteoclast formation [13,14]. During NFκB activation, the p50:P65 dimer, a subunit of NFκB, is activated and transported into the nucleus to promote the expression of NFATc1, a major osteoclast modulator that participates in the regulation of various osteoclastogenesis-related genes such as TRAP, CTK and MMP-9 [15,16,17].

Many studies have reported the inhibitory effect of fungal β (1,3), (1,6) glucan on osteoclastogenesis. Polycan, a β (1,3), (1,6) glucan from *Aureobasidium pullulans* SM-2001, protects against bone deterioration and increases the bone formation rate in ovariectomized mice [18]. Hara et al. (2021) reported that β (1,3), (1,6) glucan from *Pleurotus citrinopileatus* inhibited RANKL-induced osteoclast differentiation by supressing TRAP-positive cells [19]. Moreover, glucan from baker’s yeast (*Saccharomyces cerevisiae*) attenuated RANKL-induced osteoclastogenesis via the inhibition of NFATc1 and cFOS [20].

Therefore, various methods have been employed for reducing the molecular weight of β-glucan, including chemical (acid hydrolysis), ultrasonic disruption, thermal degradation, radiation and enzymatic methods [21,22]. Enzymatic modification has been widely used to ameliorate the functional characteristics of β-glucan because of its specifications and recyclability [23,24]. The mechanism of enzymatic degradation cuts the polysaccharide backbone into small specific oligosaccharide fragments. In addition, a previous study reported the high specification of β-1,3 glucanase to digest the β-l,3-glycosidic bond of β-glucan into dextrin or oligosaccharide [25].

Our previous study indicated that *Hevea* β-1,3-glucanase isozymes could specifically hydrolyse the particulate β-(1,3), (1,6) glucan from edible grey oyster mushroom to obtain a *Pleurotus sajor-caju* glucanoligosaccharide (Ps-GOS), which is a smaller chain, has a lower molecular weight and has a higher water solubility. Furthermore, we also examined the effect of Ps-Gos on the preosteoblastic MC3T3-E1 cell line, a bone formation cell, and results showed that Ps-GOS enhanced the proliferation, differentiation and mineralization of MC3T3-E1 osteoblasts via the bone morphogenetic protein 2 (BMP-2)/runt-related transcription factor 2 (Runx2)/MAPK/wingless-type MMTV integration site (Wnt)/β-catenin β-catenin signalling pathway [26]. Nonetheless, the effect of Ps-GOS on osteoclastogenesis has not been reported. Therefore, we aimed to investigate the biological activities and underlying molecular mechanisms of Ps-GOS on RANKL-induced osteoclastogenesis in the preosteoclastic RAW 264.7 cell line.

## 2. Results

### 2.1. Effect of Ps-GOS on Cell Viability of RAW 264.7 Cells

**Figure 1:**
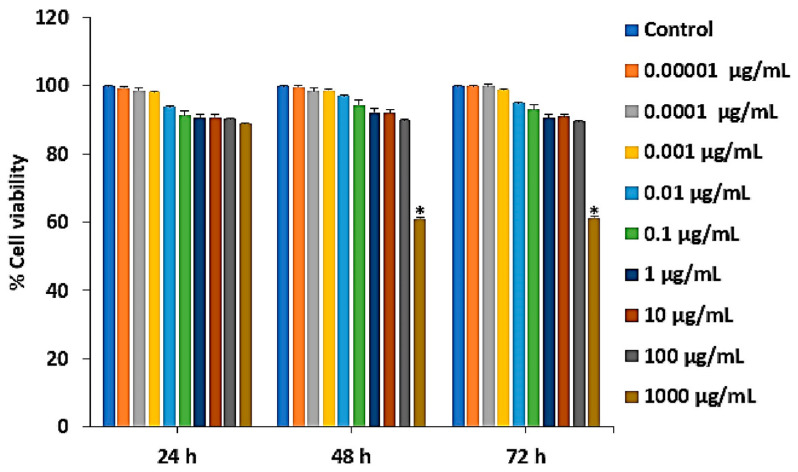
Effect of Ps-GOS on RAW 264.7 cell viability. Cells were treated with various dosages of Ps-GOS for 24, 48 and 72 h then cell viability was examined using an MTT assay. Data are shown as mean ± SEM of three experiments that were independently performed. * *p* < 0.05 compared to untreated control cells.

The cell growth of RAW 264.7 cells treated with various concentrations of Ps-GOS for 24, 48 and 72 h was examined by MTT assay. We observed that 0.00001–100 µg/mL Ps-GOS had no significant effect on cell viability at 24, 48 and 72 h. However, 1000 µg/mL Ps-GOS reduced cell viability at 48 and 72 h (Figure 1). Thus, Ps-GOS concentrations ranging from 0.01 ng/mL to 100 µg/mL had no cytotoxic effect on cells and were used in subsequent studies.

### 2.2. Effect of Ps-GOS on RANKL-induced Osteoclast Differentiation

**Figure 2:**
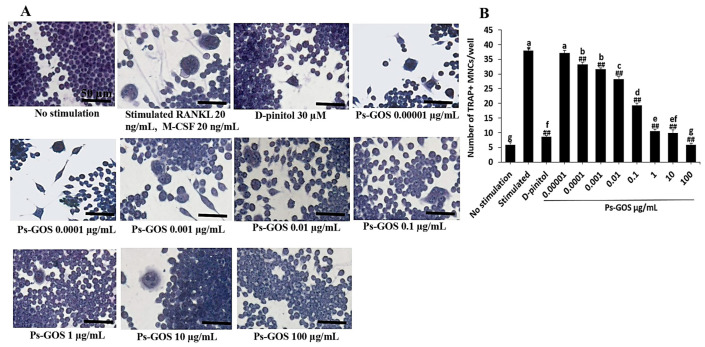
Effect of Ps-GOS on formation of multinucleated osteoclast induced by RANKL. (**A**) RAW 264.7 cells were seeded onto a 96-well plate (1.4 × 10^3^ cells/well) and separated into 4 groups: 1. No stimulation: cells were incubated in DMEM medium alone. 2. Stimulated: cells were cultured in DMEM medium + 20 ng/mL M-CSF and 20 ng/mL RANKL. 3. Experiment group: cells were incubated with Ps-Gos (0.00001–100 µg/mL) + 20 ng/mL M-CSF and 20 ng/mL RANKL. 4. Positive control: cells were treated with 30 µM of D-pinitol + 20 ng/mL M-CSF and 20 ng/mL RANKL. (**A**) TRAP staining was conducted after day 5 of incubation. (**B**) Number of TRAP-positive cells that contained three nuclei or more were counted. Results are shown as mean ± SEM of triplicated experiments that were independently performed. The columns that present different letters were significantly different at *p* < 0.05. * *p* < 0.05 vs. untreated control group. ## *p* < 0.01 vs. stimulated group that induced with RANKL (20 ng/mL) and M-CSF (20 ng/mL).

To assess the influence of Ps-GOS on RANKL-induced osteoclast differentiation, RAW 264.7 cells were treated with varying concentrations of Ps-GOS. On day 5 after incubation, stimulated group showed formation of osteoclast-like multinucleated giant cells, with about 38 ± 0.96 TRAP-positive cells (Figure 2A,B). Further, Ps-GOS treatment significantly decreased the number of TRAP-positive multinucleated cells in a dose-dependent manner. Furthermore, in the group treated with 100 µg/mL Ps-GOS, 6 ± 0.69 TRAP-positive cells were observed (Figure 2A,B). These findings showed the maximum inhibitory effect of Ps-GOS on RANKL-induced osteoclast differentiation, which was lower than that in the D-pinitol (30 µM)-treated positive control group.

### 2.3. Effect of Ps-GOS on Osteoclastic Bone Resorption In Vitro

**Figure 3:**
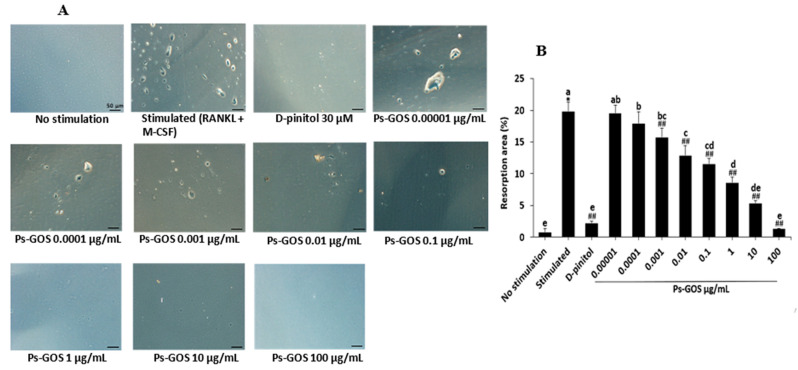
Effect of Ps-GOS on osteoclastic bone-resorptive activity. RAW 264.7 cells were plated on Osteo Assay 96-well plates and divided into the 4 groups as previously described. After 7 days, the pit formation assay was carried out. (**A**) Resorption pits were observed using a light microscope (scale bar = 50 μm) and (**B**) quantified the percentage of resorption area using ImageJ software (Version 1.53). Data are shown as mean ± SEM of three experiments that were independently performed. The columns that present different letters were significantly different at *p* < 0.05. * *p* < 0.05 vs. untreated control group. ## *p* < 0.01 vs. stimulated group that induced with RANKL (20 ng/mL) and M-CSF (20 ng/mL).

We next evaluated whether Ps-GOS could suppress osteoclastic bone-resorptive activity using the pit formation assay. As showed in Figure 3A, several pit formation areas were observed in the stimulated groups, at about 19.8% of the total area. Ps-GOS treatment markedly reduced the resorption pits in a dose-dependent manner; at the highest dose of Ps-GOS (100 µg/mL), the pit formation areas were approximately 1.36% of the total area, and this decrease was significant in comparison with the RANK-stimulated group (Figure 3B). This result indicated that Ps-GOS attenuates the bone resorption function of osteoclasts by reducing the RANKL-induced pit formation areas. 

### 2.4. Effect of Ps-GOS on Osteoclastogenic Transcription Factor Genes

**Figure 4:**
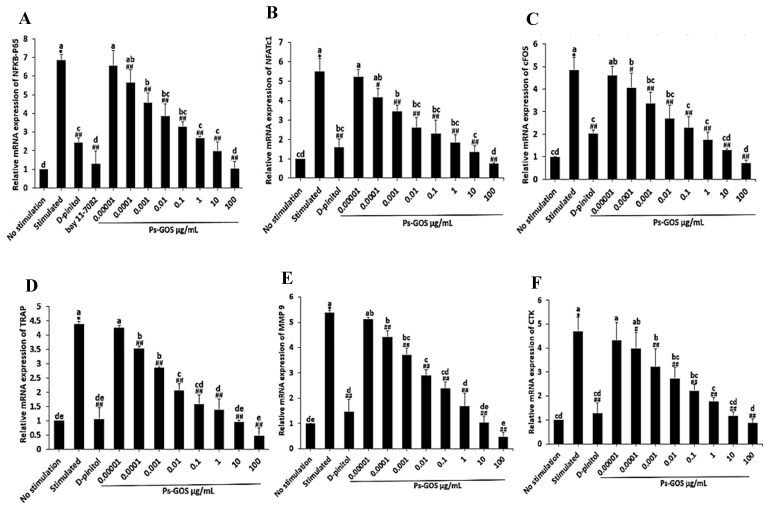
Ps-GOS suppressed mRNA expression of osteoclastogenic transcription factor genes. RAW 264.7 cells were seed onto 6-well plates and divided into 5 groups: 1. No stimulation: cells were incubated in DMEM medium alone. 2. Stimulated: cells were cultured in DMEM medium + 20 ng/mL M-CSF and 20 ng/mL RANKL. 3. Experiment group: cells were incubated with Ps-Gos (0.00001–100 µg/mL) + 20 ng/mL M-CSF and 20 ng/mL RANKL. 4. Positive control: cells were treated with 30 µM D-pinitol + 20 ng/mL M-CSF and 20 ng/mL RANKL. 5. NFκB-P65 inhibitor: cells were treated with 2.5 µM of BAY11-7082 + 20 ng/mL M-CSF and 20 ng/mL RANKL. After 3 days of incubation, the mRNA level of (**A**) NFκB-P65, (**B**) NFATc1, (**C**) cFOS, (**D**) TRAP, (**E**) MMP-9 and (**F**) CTK were evaluated using qRT-PCR. Data are shown as mean ± SEM of three experiments that were independently performed. The columns that present different letters were significantly different at *p* < 0.05. * *p* < 0.05 vs. untreated control group. # *p* < 0.05, ## *p* < 0.01 vs. stimulated group that induced with RANKL (20 ng/mL) and M-CSF (20 ng/mL).

To further explore effect of Ps-GOS on osteoclast differentiation, qRT-PCR was used to assess the mRNA levels of crucial osteoclast transcription factors NFκB-P65, NFATc1 and cFOS. As expected, stimulation greatly induced the mRNA expression of osteoclast-associated transcription factor genes NFκB-P65, NFATc1 and cFOS by approximately 6.8, 5.5 and 4.8 fold, respectively (Figure 4A–C). In contrast, Ps-GOS remarkably reduced the expression of these genes in a concentration-dependent manner (Figure 4A–C); at a concentration of 100 µg/mL, the relative expression of these modulator genes was decreased to 1.0, 0.7 and 0.7, respectively. To further investigate the inhibitory effect of Ps-GOS on NFκB activation, we treated the cells with 2.5 µM of BAY11-7082 (NFκB inhibitor) and found that the expression of NFκB-P65 in BAY11-7082-treated cells was consistent with that in Ps-GOS-treated cells (Figure 4A). These results indicated that the gene expression of osteoclastogenic transcription factor NFκB-P65 and its downstream cFOS and NFATc1 was markedly attenuated by Ps-GOS, thus proving the negative effect of Ps-GOS on NFκB-mediated osteoclastogenesis.

### 2.5. Ps-GOS Suppressed the Expression of Osteoclastogenesis-Related Genes

Next, we examined effect of Ps-GOS on the mRNA expression of osteoclastogenic marker genes using qRT-PCR. In the stimulated groups, the mRNA level of TRAP, MMP-9 and CTK were upregulated 4.3-, 5.3- and 4.7-fold, respectively. In the 100 µg/mL Ps-GOS-treated group, these genes were downregulated 0.4-, 0.4- and 0.8-fold, respectively, which was a significant decrease compared with the RANKL-stimulated group (Figure 4D–F). Thus, these results indicate that the expression of three crucial proteolytic enzymes, TRAP, MMP-9 and CTK, was markedly reduced by Ps-GOS, further confirming the suppressive effect of Ps-GOS on osteoclast differentiation and function.

### 2.6. Effect of Ps-GOS on RANKL-induced Osteoclastogenic Transcription Factor Proteins

**Figure 5:**
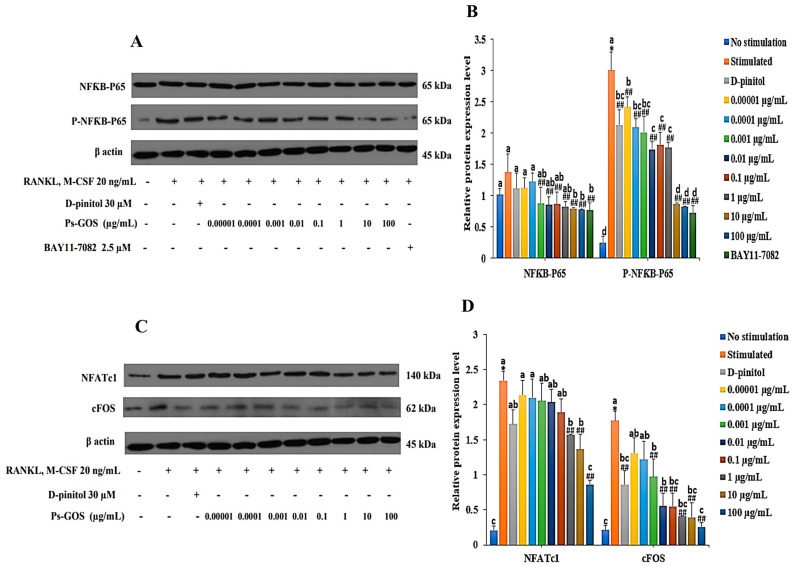
Ps-GOS downregulated protein expression of NFκB-P65 and its downstream molecules. RAW 264.7 cells were seeded onto 6-well plates and divided to 5 groups: 1. No stimulation: cells were incubated in DMEM medium alone. 2. Stimulated: cells were cultured in DMEM medium + 20 ng/mL M-CSF and 20 ng/mL RANKL. 3. Experiment group: cells were incubated with Ps-Gos (0.00001–100 µg/mL) + 20 ng/mL M-CSF and 20 ng/mL RANKL. 4. Positive control: cells were treated with 30 µM D-pinitol + 20 ng/mL M-CSF and 20 ng/mL RANKL and 5. NFκB-P65 inhibitor: cells were treated with 2.5 µM BAY11-7082 + 20 ng/mL M-CSF and 20 ng/mL RANKL. After treatment, Western blot was carried out to examine expression of (**A**) NFκB-P65 and P-NFκB-P65 and (**C**) NFATc and cFOS, and (**B**,**D**) their signal intensity was quantified using ImageJ software. Data are shown as mean ± SEM of three experiments that were independently performed. The columns that present different letters were significantly different at *p* < 0.05. * *p* < 0.05 vs. untreated control group. ## *p* < 0.01 vs. stimulated group that induced with RANKL (20 ng/mL) and M-CSF (20 ng/mL).

The vital mechanism for stimulating osteoclast differentiation and maturation is the RANKL-induced NFκB pathway. Western blot analysis showed that the protein expression level of P-NFκB-P65 was significantly suppressed by Ps-GOS in a dose-dependent manner. Furthermore, in the BAY 11-7082-treated group, phosphorylation of NFκB-P65 was markedly decreased, similar to that in the Ps-GOS-treated group (Figure 5A,B). In addition, protein expression of two pivotal osteoclast transcript factors, NFATc1 and cFOS, was significantly downregulated by Ps-GOS (Figure 5C,D). These results demonstrated that Ps-GOS markedly inhibited the expression of crucial transcription factors of mature osteoclasts not only at the mRNA level but also at the protein level, thereby providing strong evidence of its inhibitory effect on RANKL-induced osteoclast differentiation.

### 2.7. Ps-GOS Suppresses RANK Expression

**Figure 6:**
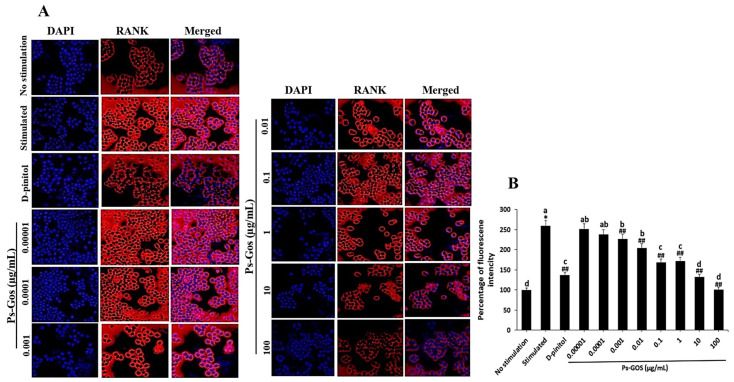
Ps-GOS significantly suppressed RANKL-induced RANK expression. RAW 264.7 cells were plated in chambered coverslips (18 wells) and separated to 4 groups: 1. No stimulation: cells were incubated in DMEM medium alone. 2. Stimulated group: cells were cultured in DMEM medium + 20 ng/mL M-CSF and 20 ng/mL RANKL. 3. Experiment group: cells were incubated with Ps-Gos (0.00001–100 µg/mL) + 20 ng/mL M-CSF and 20 ng/mL RANKL. 4. Positive control: cells were treated with 30 µM D-pinitol + 20 ng/mL M-CSF and 20 ng/mL. After 24 h of incubation, an immunofluorescence assay was carried out. (**A**) Fluorescence in the treated cells was captured using a fluorescence microscope (20× magnification) and (**B**) analysed using ImageJ software. Data are shown as mean ± SEM of three experiments that were independently performed. The columns that present different letters were significantly different at *p* < 0.05. * *p* < 0.05 vs. untreated control group. ## *p* < 0.01 vs. stimulated group that induced with RANKL (20 ng/mL) and M-CSF (20 ng/mL).

The effect of Ps-GOS on RANK expression was evaluated by an immunofluorescence assay. RANK plays a key role as a signal transmitter of osteoclastogenesis, which stimulates many osteoclast-related signalling cascades that are activated by RANKL. RANK expression was noticeably increased after RANKL stimulation for 24 h, as demonstrated in the stimulated group (Figure 6A), while RANKL-induced RANK expression was markedly suppressed by Ps-GOS. In particular, the highest Ps-GOS dosage decreased RANK expression to a level that was almost twice as low as that in RANKL-stimulated cells (Figure 6A,B). This suggested that Ps-GOS could attenuate the expression of RANK, which in turn may reduce RANKL/RANK interaction and the expression of their downstream molecules such as NFATc1 and cFOS.

## 3. Discussion

β-Glucan molecules display differences in size, structure and ability to modulate immunological factor responses. To enhance their pharmaceutical efficacy, many chemical, physical and enzymatic methods have been developed. Enzymatic modification plays a major role in the modification of the functional characteristics of polysaccharides for the purposes of depolymerization, de-esterification and debranching. Previous studies have also reported that β-glucanase could effectively reduce the molecular weight of β-glucans and enhance the solubility, functionality and bioactivity [23,27]. Duan et al. (2008) revealed that β-1,3 glucanase has a high specificity to depolymerize β-1, 3 glucan extracted from yeast (*Trichoderma* strain LE02); the obtained β-glucan had a molecular mass greater than 30 kDa and better water solubility [28].

Our previous study utilized *Hevea* β-1,3-glucanase for the enzymatic hydrolysis of *Pleurotus sajor-caju* β-glucans to obtain a lower molecular weight and higher water solubility of β-glucan oligosaccharides, Ps-GOS, which exhibits osteoblast–bone formation-enhancing activity To evaluate the potential of Ps-GOS for osteoporosis prevention, alleviation or treatment, this study aimed to investigate the inhibitory effect of Ps-GOS against osteoclastogenesis in pre-osteoclastic RAW 264.7 cells. The results revealed that Ps-GOS effectively prevented osteoporosis and is non-cytotoxic to pre-osteoclastic RAW 264.7 cells at concentrations ranging from 0.01 ng/mL to 100 µg/mL, as it did not exhibit a significant effect on cell viability at 24, 48 or 72 h.

TRAP or acid phosphatase 5 (ACP5) is the prime histochemical marker of mature osteoclasts. It plays a vital role in resorption of skeleton phosphoproteins such as sialoproteins and osteopontin [29,30]. The effect of Ps-GOS on osteoclast fusion and formation was demonstrated by the presence of TRAP-positive MNCs. We observed a large number of TRAP-positive cells in the RANKL-induced group. Moreover, Ps-GOS treatment remarkably reduced the number of TRAP-positive cells in a dose-dependent manner, suggesting that Ps-GOS inhibited the formation and fusion of mature osteoclasts, and it may also suppress bone resorptive efficacy. This is similar to a previous study showing that β-glucans from *Pleurotus citrinopileatus* inhibited RANKL-induced osteoclast formation by diminishing TRAP-positive cells [19].

Additionally, bone resorption, a unique ability of osteoclasts, was also observed in the RANKL-stimulated group. The resorption areas were decreased when treated with a low concentration of Ps-GOS. Saliently, the highest Ps-GOS dosage-treated group showed almost empty well surfaces. These findings suggested that Ps-GOS has a suppressive effect on not only osteoclast multinucleated cell formation but also their bone resorption ability. As demonstrated previously, *S. cerevisiae* β-glucan may have an inhibitory effect on bone resorption in the in vivo animal models [31].

During osteoclast differentiation, RANKL-induced NFκB was identified as an essential transduction pathway for stimulating osteoclastogenic transcription factors [32]. The upregulation of NFκB requires the activation and nuclear translocation of its subunit NFκB-P65 [33]. Thus, to confirm the effect of Ps-GOS on RANKL-induced NFκB expression, we assessed the expression of NFκB-P65 at both the mRNA and protein levels. We found that RANKL activated NFκB-P65 expression compared with no stimulation, while Ps-GOS treatment effectively suppressed the expression of NFκB-P65 at both the transcriptional and translational levels. To further validate the inhibitory effect of Ps-GOS, cells were treated with BAY11-7082, an NFκB-P65 inhibitor. As expected, NFκB-P65 expression was suppressed by its inhibitor, and the results were consistent with those observed after Ps-GOS treatment.

Furthermore, the effects of Ps-GOS on cFOS and NFATc1, the major modulators of osteoclast development, were also evaluated. NFATc1 is widely known to regulate several osteoclastogenesis-related genes, including TRAP, CTK, MMP-9 and the calcitonin receptor; however, the activation of NFATc1 requires the essential cooperator c-FOS [34]. In our present study, we found that Ps-GOS inhibited the expression of NFATc1 and cFOS, which may consequently attenuate the expression of osteoclastogenesis-related genes. This was consistent with recent work showing that glucan from baker’s yeast (*Saccharomyces cerevisiae*) inhibited RANKL-induced osteoclastogenesis via the suppression of NFATc1 and cFOS [20]. A previous in vivo study revealed the osteoporotic bone phenotype in NFATc1-deficient mice due to a defect in osteoclast differentiation [35]. Moreover, cFos-null mice developed osteopetrosis owing to the absence of osteoclast lineage commitment [36]. All of this evidence suggests that the activation of NFκB and c-FOS is required for the recruitment of NFATc1 [37,38].

The stimulation of NFATc1 in osteoclast precursors contributes to the ability of mature osteoclasts by governing the osteoclast-specific genes [34,39]. As mentioned, TRAP digests phosphoproteins [29,30], MMP-9 is a gelatinase that promotes osteoclast differentiation [33,40], and CTK possesses the crucial ability to digest organic matrices, such as collagen type I [41]. The reduction of proteolytic enzymes, including TRAP, MMP-9 and CTK, is considered a determinant of osteoclast-resorption activity and was observed in the present study.

The RANK/RANKL/osteoprotegerin (OPG) axis plays a role in regulating bone remodelling and osteoclastogenesis. RANKL and OPG are generated by osteoblasts [42]. Binding of RANKL to RANK activates osteoclast differentiation, while OPG, a decoy receptor, interferes with RANK/RANKL interaction resulting in the suppression of osteoclastogenesis [43,44]. Since RANK is the initial signal transmitter activating many subsequent osteoclast signalling cascades, we, therefore, detected the effect of Ps-GOS on RANKL-induced RANK expression. Interestingly, the stimulated group demonstrated a markedly increased RANK expression. Moreover, Ps-GOS treatment markedly reduced this increase in a dose-dependent manner, which may imply the inhibition of RANK/RANKL interaction and subsequent essential modulators that are obligatory for osteoclasts. Supporting this, the expression of master transcription factors, such as NFATc1 and cFOS and their downstream molecules, such as TRAP, CTK and MMP-9, were significantly decreased. This further indicated the suppressive effect of Ps-GOS on osteoclast differentiation and function.

To the best of our knowledge, this study is the first to report that low molecular weight, highly water-soluble Ps-GOS suppresses osteoclast differentiation and bone resorption, which are accompanied by the inhibition of TRAP, CTK and MMP-9 expression through the attenuation of the RANK/NFκB/cFOS/NFATc1 pathway. Our study is consistent with several reports demonstrating inhibitory effects of β-glucans from various sources, including glucan from baker’s yeast and curdlan (low MW), on osteoclast differentiation via the suppression of NFATc1 and activation and RANKL expression [20,45,46].

Together with our previous study findings, we established the stimulatory effect of Ps-GOS on osteoblast proliferation, differentiation and mineralization. Ps-GOS acts as a potential modulator of bone homeostasis by enhancing osteoblast-bone formation and suppressing osteoclast–bone resorption.

However, this study has a potential limitation. The investigation was solely conducted using the secondary cell line, RAW 264.7, which is a commonly accepted model for studying osteoclastogenesis. Further exploration of its effects on primary cell lines, such as bone marrow-derived macrophages (BMM), is warranted. Moreover, to gain deeper insights into the efficacy of Ps-Gos on bone metabolism, its effects on ovariectomized models and clinical studies should be explored. Consequently, Ps-Gos may potentially be developed as a candidate for adjunct therapy or preventive agents for osteoporosis.

## 4. Materials and Methods

### 4.1. Materials

Dulbecco’s Modified Eagle’s Medium (DMEM, No. 11965092) and cell culture reagents were obtained from Gibco (Grand Island, NY, USA). D-pinitol (No. 441252-100MG) and the TRAP staining kit (No 387A-1KT) was purchased from Sigma Aldrich (St. Louis, MO, USA). Recombinant RANKL (No. 462-TEC-010) and M-CSF (No. 416-ML-010) were purchased from R&D Systems (Minneapolis, MN, USA). All specific primers were synthesized by Macrogen (Seoul, Korea). Antibodies for NFATc1 (No. 8032S), cFOS (No. 2250S), NFκB-P65 (No. 8242S), P-NFκB-P65 (No. 3033S) and β-actin (No. 4970S) were obtained from Cell Signaling Technology (Danvers, MA, USA). Antibodies for RANK (No. sc-390655) was provided by Santa Cruz Biotechnology (Dallas, TX, USA). Ps-GOS was received from the Centre for Natural Rubber Latex Biotechnology Research and Innovation Development; its extraction and purification method were detailed in a previous study [26]. All other reagents and solvents used were supplied by a local company and were of analytical grade.

### 4.2. Cell Culture and Mature Osteoclast Induction

The murine RAW264.7 cell line, which has been widely used as a cell model for studying osteoclastogenesis, was obtained from the American Type Culture Collection (ATCC, Manassas, VA, USA). These cells have special characteristics, such as being readily accessible, sensitive and quick to differentiate into active osteoclasts. They demonstrate homogenization of osteoclast progenitors and are easily cultured and passed [47]. The cells were cultured in completed DMEM medium (10% fetal bovine serum (FBS), streptomycin (100 µg/mL) and penicillin (100 units/mL)) at 37 °C and 5% CO_2_. To induce mature osteoclast formation, the cells were treated with RANKL (20 ng/mL) and M-CSF (20 ng/mL) for 5 days. The cultured medium was changed every 2 days.

### 4.3. Cell Viability Assay

To examine effect of Ps-GOS on cell viability, RAW 264.7 cells at a density of 5 × 10^3^ cells/well were seeded onto a 96-well plate. After cell attachment (overnight incubation), the cells were then incubated with various doses of Ps-GOS (0.00001, 0.0001, 0.001, 0.01, 0.1, 1, 10, 100 and 1000 µg/mL) for 24, 48 and 72 h. After incubation, a 3-(4,5-dimethylthiazol-2-yl)-2,5-diphenyltetrazolium bromide (MTT) assay was performed. Then, formazan was dissolved in 50 mL of dimethyl sulphoxide (DMSO). Finally, we detected the optical density (OD) of each well using a microplate reader at 570 nm (Bio-tek Instruments, Winooski, VT, USA).

### 4.4. Osteoclast Differentiation Assay

TRAP staining was conducted to evaluate the effect of Ps-GOS on osteoclast differentiation. First, the cells (1.4 × 10^3^ cells/well) were seeded in a 96-well plate and divided into the following 4 groups: 1. No stimulation: cells were incubated in DMEM medium alone. 2. Stimulated: cells were cultured in DMEM medium + 20 ng/mL M-CSF and 20 ng/mL RANKL. 3. Experiment group: cells were incubated with Ps-Gos (0.00001–100 µg/mL) + 20 ng/mL M-CSF and 20 ng/mL RANKL. 4. Positive control: cells were treated with 30 µM D-pinitol + 20 ng/mL M-CSF and 20 ng/mL RANKL. After 5 days of incubation, the cells were fixed with 4% paraformaldehyde and stained with the TRAP staining kit according to the manufacturer’s instructions. TRAP-positive multinucleated cells containing at least three nuclei were counted as mature osteoclasts using a light microscope (Olympus, Tokyo, Japan).

### 4.5. Pit formation Assay

To evaluate the effect of Ps-GOS on bone resorptive activity, RAW 264.7 cells (1.4 × 10^3^ cells/well) were seeded onto Osteo Assay surface 96-well plates (No. CLS3988, Corning osteoassay, Glendale, AZ, USA) and divided into 4 groups as described in the osteoclast differentiation assay. After 7 days of treatment, the cells were incubated for 5 min with bleach solution (10%) and washed with distilled water. Finally, the pit formation areas were observed using a light microscope (Olympus, Tokyo, Japan) and analysed with ImageJ software.

### 4.6. Quantitative Real-Time Polymerase Chain Reaction (qRT-PCR)

RAW 264.7 cells were plated (1 × 10^5^ cells/well) into 6-well plates and divided into the following 5 groups: 1. No stimulation: cells were incubated in DMEM medium alone. 2. Stimulated: cells were cultured in DMEM medium + 20 ng/mL M-CSF and 20 ng/mL RANKL. 3. Experiment group: cells were incubated with Ps-Gos (0.00001–100 µg/mL) + 20 ng/mL M-CSF and 20 ng/mL RANKL. 4. Positive control: cells were treated with 30 µM D-pinitol + 20 ng/mL M-CSF and 20 ng/mL RANKL. 5. NFκB-P65 inhibitor: cells were treated with 2.5 µM BAY11-7082 + 20 ng/mL M-CSF and 20 ng/mL RANKL. After 3 days of treatment, total RNA was separated using TRIzol reagent (No. 15596026, Thermo Fisher Scientific Inc., Waltham, UT, USA) according to the manufacturer’s indications. Subsequently, cDNA was synthesized by reverse transcription. qRT-PCR was carried out using 5×HOT FIREPol^®^Blend master mix (No. 04-27-00125, Solis Biodyne, Tartu, Estonia), using the following protocol: 95 °C for 15 min for initial denaturation of template cDNA, 40 cycles of denaturation (94 °C for 15 s), annealing (57 °C for 30 s) and final elongation (72 °C for 30 s). The specific primers are displayed in Table 1, and the expression of the housekeeping gene *GAPDH* was used for normalization. Relative mRNA expression of osteoclastogenic marker genes was calculated by the 2^−ΔΔCt^ method [20].

### 4.7. Western Blot Analysis

To examine osteoclastogenic protein expression affected by Ps-GOS, RAW 264.7 cells (5 × 10^5^ cells/well) were plated on a 6-well plate and divided into the 5 group mentioned in the qRT-PCR assay section. After incubation for 3 days, the cells were lysed with radioimmunoprecipitation assay (RIPA, No. 89900) buffer containing a phosphatase inhibitor cocktail (No. 78420) and proteinase inhibitor cocktail (No. 87786, Thermo Fisher Scientific Inc., Waltham, UT, USA), and consequently, concentrations of protein were measured using a bicinchoninic acid protein assay kit (BCA) (No. 23225, Thermo Fisher Scientific Inc., Waltham, UT, USA). An equal amount of protein was loaded onto a 12% acrylamide gel and separated using sodium dodecyl sulphate-polyacrylamide gel electrophoresis (SDS-PAGE). Separated samples were then electrotransferred to polyvinylidene difluoride membranes (No. IPVH85R, Millipore, Jaffrey, NH, USA). Then, membranes were incubated for 1 h with 5% non-fat dried milk for blocking of non-specific and incubated with a specific primary antibody (1:1000; Cell Signaling Technology) at 4 °C overnight with continuous shaking, followed by washing with tris-buffered saline with Tween 20 (TBST) three times and incubated with horseradish peroxidase-conjugated secondary antibodies (1:5000; No. 7074S, Cell Signaling Technology) for 1 h. The signals were developed using an ECL Substrate Kit (No. 32209) and then exposed to an X-ray film (No. 34089, Thermo Fisher Scientific Inc., Waltham, UT, USA). The films were scanned and analysed using ImageJ software.

### 4.8. Immunofluorescence Staining

RAW 264.7 cells were plated in a chambered coverslip (18 wells) at a density of 2 × 10^4^ cells/well and separated into the following 4 groups: 1. No stimulation: cells were incubated in DMEM medium alone. 2. Stimulated: cells were cultured in DMEM medium + 20 ng/mL M-CSF and 20 ng/mL RANKL. 3. Experiment group: cells were incubated with Ps-Gos (0.00001–100 µg/mL) + 20 ng/mL M-CSF and 20 ng/mL RANKL. 4. Positive control: cells were treated with 30 µM D-pinitol + 20 ng/mL M-CSF and 20 ng/mL RANKL. After incubation for 24 h, cells were fixed with 4% paraformaldehyde and permeabilized with 0.1% Triton X-100 (No. 39487. Cell Signaling Technology, Danvers, MA, USA). After blocking with 5% goat serum (S26-100ML, Sigma Aldrich), they were incubated with the primary anti-mouse RANK antibody (1: 500; sc-390655, Santa Cruz Biotechnology) at 4 °C overnight with shaking. Subsequently, the cells were incubated with a secondary antibody (1:200; No. sc-516141, Santa Cruz Biotechnology) for 3 h and counterstained with 4′,6-diamidino-2-phenylindole (DAPI) (No. 4083, Cell Signaling Technology, Danvers, MA, USA) for 5 min. Finally, mounting medium was carefully added to preserve the fluorescence signal, and photographs were taken with a fluorescence microscope (Olympus, Tokyo, Japan). The fluorescence intensity was analysed with ImageJ software.

### 4.9. Statistical Analysis

All data are displayed as mean ± standard error of mean (SEM) of a triplicated experiment. Each experiment was independently performed and resulted in similar results. Results were analysed by SPSS 23 statistical software (SPSS Inc., Chicago, IL, USA). Statistical differences were compared by one-way analysis of variance (ANOVA) followed by Tukey’s and Duncan’s multiple range test. In all cases, *p* < 0.05 was considered statistically significant.

## 5. Conclusions

This is the first report showing the inhibitory effect of Ps-GOS on osteoclastogenesis. The underlying mechanism involves blocking RANK and downregulation of the NFκB-induced cFOS and NFATc1 pathways, resulting in the suppression of TRAP, CTK and MMP-9. Ps-GOS exhibits potential as a promising natural compound for mitigating and averting osteoporosis. Nevertheless, additional exploration using an in vivo model and clinical trials is imperative.

## Figures and Tables

**Table 1:** Primer sequences of qRT-PCR.

Gene	Sequence	GenBank Accession No.
NF-κB-P65	F: TCACCGGCCTCATCCACAT	XM_006531694.4
	R: TGGCTAATGGCTTGCTCCAG	
NFATc1	F: CACACACCCCGCATGTCA	NM_001164110.1
	R: CGGGCCGCAAAGTTTCTC	
TRAP	F: TGGATTCATGGGTGGTGCTG	XM_006509946.3
	R: CGTCCTCAAAGGTCTCCTGG	
c-Fos	F: AGCTCCCACCAGTGTCTACC	NM_010234.3
	R: TCACCGTGGGGATAAAGTTGG	
Cathepsin K	F: AGTAGCCACGCTTCCTATCC	NM_007802.4
	R: GAGAGGCCTCCAGGTTATGG	
MMP-9	F: CTCTGCTGCCCCTTACCAG	NM_013599.5
	R: CACAGCGTGGTGTTCGAATG	
GAPDH	F: AGGTCGGTGTGAACGGATTTG	XM_036165840.1
	R:TGTAGACCATGTAGTTGAGGTCA	

NF-κB-P65, nuclear factor NF-kappa-B P65; NFATc1, nuclear factor of activated T cell cytoplasmic 1; TRAP, tartrate-resistant acid phosphatase; c-Fos, Fos proto-oncogene; MMP-9, matrix metallopeptidase 9; GAPDH, glyceraldehyde 3-phosphate dehydrogenase.

## Data Availability

Data are contained within the article.
